# What Shall I Eat?

**Published:** 1911-07

**Authors:** 


**What Shall I Eat? A Manual of Rational Feeding.** By **Dr. F. X. Gouraud,** Formerly Chief of the Laboratory of the Medical Faculty of Paris. With a Preface by Prof. Armand Gautier, of Paris. Only authorized translation by Francis J. Rebman. Octavo, pp. 379. New York: Rebman Co. 1911. (Cloth, $1.50.)

In this age of "Therapeutic or Drug Nihilism," dietetics is assuming its proper place in the treatment of disease. Naturally the subject has been studied very fully by the physiologic chemist, and has resulted in the proper understanding of caloric values, the exact composition of foods and the metabolic processes of the human economy.

This book is the result of such study. Its author treats his subject clearly, concisely and withal practically. Each article of food is discussed, its chemical composition stated, its food value discussed and the indications as well as the contraindications in the normal state of health and disease given in every instance. The reader, is he a physician, has an excellent guide before him for use in outlining dietary for his patients.

With a little study, the average practitioner unfamiliar with the determination of caloric values can easily master this subject of dietetics; nor is the work too technical for the trained nurse, dietetician or intelligent layman.

The chapters on alcohol, vegetarianism and wheat bread are particularly interesting and valuable to all. L. K.

